# Data Privacy Concerns Using mHealth Apps and Smart Speakers: Comparative Interview Study Among Mature Adults

**DOI:** 10.2196/28025

**Published:** 2022-06-13

**Authors:** Tanja Schroeder, Maximilian Haug, Heiko Gewald

**Affiliations:** 1 Centre for Health Systems and Safety Research Australian Institute of Health Innovation Macquarie University Sydney Australia; 2 Center for Research on Service Sciences Neu-Ulm University of Applied Sciences Neu-Ulm Germany

**Keywords:** data privacy concerns, privacy paradox, mHealth app, smart speaker, mature adults, smartphone

## Abstract

**Background:**

New technologies such as mobile health (mHealth) apps and smart speakers make intensive use of sensitive personal data. Users are typically aware of this and express concerns about their data privacy. However, many people use these technologies although they think their data are not well protected. This raises specific concerns for sensitive health data.

**Objective:**

This study aimed to contribute to a better understanding of data privacy concerns of mature adults using new technologies and provide insights into their data privacy expectations and associated risks and the corresponding actions of users in 2 different data contexts: mHealth apps and smart speakers.

**Methods:**

This exploratory research adopted a qualitative approach, engaging with 20 mature adults (aged >45 years). In a 6-month test period, 10 (50%) participants used a smart speaker and 10 (50%) participants used an mHealth app. In interviews conducted before and after the test period, we assessed the influence of data privacy concerns on technology acceptance, use behavior, and continued use intention.

**Results:**

Our results show that although participants are generally aware of the need to protect their data privacy, they accept the risk of misuse of their private data when using the technology. Surprisingly, the most frequently stated risk was not the misuse of personal health data but the fear of receiving more personalized advertisements. Similarly, surprisingly, our results indicate that participants value recorded verbal data higher than personal health data.

**Conclusions:**

Older adults are initially concerned about risks to their data privacy associated with using data-intensive technologies, but those concerns diminish fairly quickly, culminating in resignation. We find that participants do not differentiate between risky behaviors, depending on the type of private data used by different technologies.

## Introduction

### Overview

A mobile health (mHealth) app is a specific type of digital health app that uses mobile devices such as smartphones and tablets that are already integrated into daily lives of people. People use mHealth apps to monitor their health or access medical information or assistance through wireless mobile devices such as smartphones and portable monitoring devices [1]. Similarly, mHealth apps enable health care providers to monitor certain user activities and behaviors so that they can provide personalized health care advice. Other technologies can also be used to support personalized health care support. For example, smart speakers with artificial intelligence–operated assistants that help users more easily access information or control other devices via their voice can be used for this purpose [2,3]. Although smart speakers are often used for everyday activities, such as playing music, setting a timer, or hearing a weather report, they can also be used to remind users to take their medication or answer health-related questions. Smart speakers collect and process a variety of private information and are typically used in private environments. Because a smart speaker must be able to recognize the voice activation keyword at any time, its microphone’s default status is active. Therefore, many people associate smart speakers with involuntary personal information disclosure [4].

The increasing use of digital and mobile technologies, combined with the need for personalized and cost-efficient health care, has fostered the emergence of mHealth technologies. Such technologies have many potential health care benefits, such as the ability to monitor users’ health status continuously and remotely, increased diagnostic accuracy, earlier awareness of new problems, lower health care costs, greater availability of health care to people living in remote areas, and improved doctor-patient communications.

Despite these potential benefits, the sensitivity of individuals’ private data collected and recorded by these digital apps raises concerns about the privacy of this information [5,6]. For example, some potential users want to control what people in their private environment, such as family members, know about their health status, perhaps because they fear being judged, reprimanded, discriminated against, or even penalized for their physical and health status [7]. Some people may not want to take care of their family members because of their current health status [7,8]. The fear of social stigmatism is another reason people may not want others to know their health status [8]. This may apply to physical or mental disabilities, mental illnesses, or certain diseases such as HIV and Alzheimer [8,9].

In this study, we define *privacy* as the right of individuals, groups, or institutions to determine when, how, and to what extent information about them is shared with others [10]. Privacy is a subjective concept linked to an individual’s perception of what constitutes a threat to their personal property or physical or moral integrity, depending on cultural aspects and sociodemographic issues [11]. Users’ perspectives on interactions and communications influence their data privacy–related decisions on a range of privacy issues, including technical issues such as regulating visibility in social networks and using smartphone apps that collect confidential data [12].

Most extant data privacy literature focusing on mHealth deals with the technical aspects of privacy, such as the level of security of information transmitted over mobile networks and stored on a device or in a cloud service needed to prevent unauthorized access to a patient’s information [8,13-15]. However, privacy is not only a technical issue. For example, the collaborative use of mHealth apps for shared care management imposes other privacy requirements related to human factors, such as the wishes and preferences of the user when exchanging health information with authorized institutions and external persons such as professional health care providers [16].

Extant research shows that privacy concerns are a major inhibitor of the adoption and use of both mHealth apps and smart speakers [4,17]. Because the 2 technologies access different types of sensitive personal data, potential users may have different privacy concerns regarding each technology. To investigate this issue, we posed the following research questions (RQs):

1. RQ1: What privacy concerns do potential users associate with mHealth apps and smart speakers?
2. RQ2: What data privacy–related risks do potential users attribute to the use of mHealth apps and smart speakers?
3. RQ3: What privacy-related issues lead to rejection of mHealth apps and smart speakers?

Although the use of smart speakers is roughly equal among *mature adults*, which we define as people who are aged >45 years and adults aged <45 years [18], extant research shows that mobile apps for health care purposes are most commonly used by mature adults [19]. Because this study addresses both technologies, we focus on the mature adult user group, providing 50% (10/20) of the participants with a smart speaker and 50% (10/20) of the participants with an mHealth app to use in a 6-month testing phase. All the participants were interviewed before and after the testing phase.

### Background

One of the main reasons why people are reluctant to use mHealth apps is concerns about the security and privacy of their health-related data [20-23]. Users often do not know what kind of data mHealth apps collect and store and who can access data entered manually or collected by sensors and for what purposes [20,24]. Studies show that users have greater security and privacy concerns about mHealth apps that focus on issues associated with stigmatization, discrimination, or social isolation, such as sexually transmitted diseases, sexual orientation, and mental illnesses [25-29]. Considering that millions of patients’ health data have been compromised through hacking or other incidents in recent years, these concerns are valid [30]. Despite private data security breaches, few mHealth apps have security features that adequately protect users’ private health data [31-33].

### Privacy Theories

Extant research has not yet fully explored the specific role of privacy concerns in the acceptance and use of technology. The privacy calculus theory [34,35] and the so-called *privacy paradox* [36] are central concepts in privacy research. They illustrate the ambivalent influence of privacy on behavior.

The privacy calculus theory assumes that individuals engage cognitively to weigh the perceived costs and benefits of a behavior [34,35]. If the benefits outweigh the potential harm that privacy abuse can induce, individuals engage with the technology. However, this view is partly challenged by the privacy paradox, a phenomenon in which individuals engage with a technology even though the privacy concerns they associate with using the technology outweigh the anticipated benefits of using it [36,37]. Although both phenomena are well documented in practice, technology adoption scholars have yet to explain this conundrum [38].

### Privacy Research

Computer system privacy has long been a concern. In 1969, a study by Hoffman [39] discussed strategies for user access control and data protection, emphasizing the need to weigh the efficiency benefits of storing personal information against the risks of third-party access to such information. The study examined legal and administrative safeguards to protect sensitive information on computers and evaluated the technical solutions available at the time.

More recently, a study by Barth and De Jong [40] focused on privacy-related human factors. They conducted a systematic literature review to understand the web-based privacy paradox, where users indicate great concern about the privacy of their personal information but do very little to protect such data. The authors identified 35 theoretical approaches to decision-making and identified different perspectives on the paradox. Specifically, they discuss the decision-making process after rational or irrational risk-benefit calculations in the specific context of the privacy paradox.

Pavlou [41] described the data protection paradox in his privacy briefings as a phenomenon in which individuals express strong concerns about their privacy but behave in a way that contradicts these concerns. For example, some consumers still share their personal information despite privacy concerns. Even after considering the user perspective, Aïmeur [42] addressed the question of how to achieve a good compromise between privacy and user personalization. He mentioned that an increasing number of users can only control their data by fine-tuning the app settings. The author also argued that mHealth would benefit significantly if users had direct control over when, where, and with whom their personal data were shared.

### Privacy-Personalization Paradox

Varshney [43] described privacy as the right of a group or individual to isolate or retain information about themselves. Personalization technologies offer users a wide range of services from which to choose but also require users to disclose more personal information, which may raise privacy concerns [44]. This has been compounded by the emergence of smartphones that can capture personal information more accurately [45]. For example, health care counseling provided via mobile platforms can reduce the need for personal interaction, but app users must then share information relevant to their health, such as health status, preferences, and lifestyle, as well as their telephone number with service providers to use personalized health care counseling services that overcome geographic barriers and save time. This, in turn, raises privacy concerns regarding the collection of sensitive consumer information: a technological paradox [46]. Although consumers want personalized services, they are reluctant to disclose personal information and want to disclose as little information as possible.

### Demographics

Demographic differences between potential consumers are linked to behavioral intentions [47-50]. Some studies have focused on age differences in technology adoption, suggesting that there are differences in intentional behavior among different age groups [51]. However, Featherman and Pavlou [52] found that the validity of theoretical constructs, including models of health behavior change, is not well documented at all life stages. Most scholars agree that as people age, their physical and mental activities change, which affects their health status and decision-making [53]. Researchers have recently recognized that studying age differences in behavioral intention in the health context is both useful and essential. Ziefle and Röcker [54] found that age differences played an important role in the acceptance of health-related technologies. Similarly, Sintonen and Immonen [55] found that older participants’ intention to adopt technology varies over time and according to the service provided, whereas a study by Guo et al [56] found that *dark-side* constructs influence older participants’ intention to adopt mHealth services.

## Methods

### Recruitment and Demographics

This is an exploratory qualitative study with 20 participants divided into 2 groups. Semistructured interviews were conducted between October 2019 and April 2020, in southern Germany. Each group consisted of 10 participants aged between 46 and 80 years. Group A participants used smart speakers with Amazon Alexa technology, and group B participants used a self-provided mHealth app on their Android smartphone. Both technologies were provided to the participants free of charge.

Smart speakers are voice-controlled, enabling users to play music or news, access information, place telephone calls, and perform other tasks via voice commands. The smart speaker is activated by speaking a predefined voice command. The smart speakers have several levels of built-in privacy measures. For example, a microphone can be deactivated by pushing a button that interrupts its power supply. Users also retain full control over voice recordings. Users can also control whether, when, and which voice recordings are accessed or accessible by third parties through the internet.

The mHealth app incorporates a health diary that users can use to track their daily health status, water intake, and other health-related information. This app is mainly intended to make daily life easier for older people. Referring to a digital health diary should allow health care providers to collect relevant information more effectively and quickly and make the process less burdensome for older people. In addition, it aims to prevent a decline in cognitive performance. Using the *My values* feature, users can choose from a wide range of functions within the app, including body temperature, blood pressure, heart rate, blood glucose levels, and weight. Users can query the recorded data using various time intervals. In addition, users can activate a push notification function to receive reminders to enter current measurement values.

The participants were recruited in southern Germany through flyers and posters in senior citizen centers and postings on social media. Participation was strictly voluntary, and no incentives were provided. All participants interviewed were informed of the research team’s data protection arrangements and signed a document to this effect in full compliance with the European Union data protection regulations, the General Data Protection Regulation (GDPR). Participants interested in taking part in the study were given a participant information sheet and an informed consent form detailing the participation requirements in advance, with the option to have the form explained if necessary. Participants signed and returned the form, indicating their informed consent. The consent forms were retained and stored securely as a record of informed consent. Participants could withdraw from the interviews at any time during the study if they no longer wanted to be included. Furthermore, the researchers were available to answer questions at any time. The research group saved all participant data anonymously and retained all written and audio materials collected during the interviews until the end of the data retention period. All project data are stored electronically on secure password-protected servers and accessible only by designated and approved project team members. All the data will be destroyed at the end of the data retention period.

Table 1 provides demographic information of the participants.

All participants were considered IT affine, demonstrating interest in digital technologies such as smartphones, tablets, PCs, smart watches, or smart speakers. All participants actively used a smartphone and at least one social media service regularly and demonstrated at least an average internet use intensity for their age group. All interviewees were able to install and set up a smart speaker or an mHealth app with little or no assistance.

**Table 1:** Demographics of the participants (N=20).

Participant group and informant			Age (years)		Gender
**Group A**					
	A1	56		Female	
	A2	59		Female	
	A3	54		Female	
	A4	48		Female	
	A5	49		Male	
	A6	70		Male	
	A7	68		Male	
	A8	56		Male	
	A9	54		Male	
	A10	46		Male	
**Group B**					
	B1	52		Male	
	B2	50		Male	
	B3	55		Female	
	B4	60		Female	
	B5	56		Female	
	B6	61		Female	
	B7	54		Female	
	B8	52		Female	
	B9	80		Male	
	B10	51		Male	

### Data-Gathering Process

The study was structured into three phases: (1) before, (2) during, and (3) after testing the respective technologies.

Phase 1 began with participant recruitment and ended with the delivery of the technology to be tested. This phase included the first conversation to give participants detailed information about the study approach and timeline, collect demographic data, interview all participants about their expectations toward using new technology and their concerns about protecting their private data, and ask participants who would test the mHealth app about their experiences in preventive health care. We also asked specific questions about privacy concerns related to the technology tested in our study.

Phase 2 started with the delivery of the technology and ended when the technology was returned (smart speaker) or uninstalled (mHealth app). This 6-month phase gave participants ample time to test the technology thoroughly. During this phase, research assistants (MH, Jennifer Klaus, and Jaro Lanza) were available to help if participants encountered difficulties using the technology.

Phase 3 started when the technology was returned or uninstalled and ended when all data were collected and ready to be analyzed. During this phase, we conducted posttest interviews with each participant to discuss their use behavior, observations, problems, and concerns. We paid special attention to how their assessment of data privacy concerns changed over time and how this influenced their intention to continue using the technology.

### Interview Structure and Data Analysis Method

In phases 1 and 3, we conducted individual semistructured interviews with each participant, following an interview guide and drawing on a list of topics and specific questions. We posed open-ended questions that allowed the respondents to explore their experiences and views. The interview guide helped focus the interview and ensured the comparability of data collected among multiple participants in various interview settings and by different researchers. The interview process was systematic and comprehensive, but the interviewer was free to follow up on issues of greater interest or importance to the participant during the interview by adapting preformulated questions ad hoc to gain a deeper and more holistic understanding of the participants’ perspectives and perceptions.

As the aim of this study was to understand the relationships among privacy concerns, risk perception, and use behavior in the technology-use context, we focused principally on privacy concerns, the impact of data misuse, and termination of use.

The 10 pretest and the 10 posttest interviews lasted 15 to 60 minutes each (average 35, SD 15.59 minutes) and were conducted face to face or via telephone or videoconference. The interviews were conducted in German and recorded, transcribed, and coded using an open coding approach using NVivo (version 10; QSR International) software independently by 2 research team members (TS and MH). The research team coded the data parallel to data collection. Subsequently, the data were triangulated according to the recommendations by Miles et al [57] and Flick [58]. The analysis took an inductive and interpretative approach. The inductive approach is a systematic procedure for analyzing qualitative data in which the analysis is guided by specific objectives [59].

In the second round of analysis, the codes from the individual interviews were correlated [57] to cast light on the specific characteristics of these topics and the influence of these factors in the context of the two technologies.

### Ethics Approval

This study did not require ethical approval according to the guideline of the applicable Ethics Committee of the Bavarian Universities (Gemeinsame Ethikkommision der Hochschulen Bayerns [60]), as no risks or harm to the participants were expected and the basic ethical principles were not violated. All participants received a participant information and consent form explaining the requirements for participation, with the option to have the form explained to them if needed and gave their verbal consent as a sign of informed consent if they were willing to participate at the time of the interview. They were also given the opportunity to complete and sign the participant consent form to indicate their agreement to the interview being conducted.

## Results

### Overview

The pretest interviews focused on participants’ general expectations regarding the technology, as well as their privacy concerns and expected risks associated with using the technology. The posttest interviews focused generally on how the informants had used the technology during the test phase, specifically on whether their privacy concerns and their perceptions of the risks changed during the 6-month testing phase, and if the intention to continue using the technology.

To better segregate the 2 different types of data used by the technologies we refer to “health data” for the data used by the mHealth app which predominantly consist of the participant’s health status or well-being information and “personal data” for the data collected by the smart speaker, which comprises mainly intended and unintended speech, as well as the information transmitted when the user gives commands to the speaker.

### Privacy Concerns

#### Overview

Individuals’ privacy concerns are shaped and influenced by many factors, such as personal experiences, media coverage, and the social environment [61-64]. Therefore, it is essential to understand participants’ perceptions of individual data privacy–related issues to be able to classify their statements and derive results. To better understand how privacy concerns impact the use of smart speakers and mHealth apps, we asked participants directly about their privacy concerns and perceptions of privacy-related issues.

It should be noted that some of the participants testing a smart speaker had previous experience using them, were more aware of privacy-related issues associated with them, and had already formed opinions about the risks and benefits of using them. In contrast, the participants testing a smart speaker for the first time had a basic understanding of the technology but were less aware of the privacy-related issues associated with them and did not know what to expect. None of the participants testing the mHealth app had prior experience of using an mHealth app.

The following 2 sections present our core findings, supported by exemplary quotations translated from the German original. The alphanumeric codes after each quotation refer to the quoted participants and other participants who expressed similar viewpoints.

#### Smart Speakers

Participants commonly expressed awareness of privacy issues associated with using a smart speaker:

I would say that I have a high level of data protection awareness. [...] I am very aware that the data that I enter on the Internet or that is processed via the Internet can be accessed by providers and misused.

A8; A10

Most (8/10, 80%) participants expressed concerns regarding data protection:

No, I do not think that the data is secure.

A2; A5; A8; A9

I do not think the data is secure. The data is recorded and stored, and once the data is on the Internet, it is not safe for me either.

A3

In addition, most (6/10, 60%) participants articulated some degree of concern that their data would be stored somewhere unknown and used without their permission. This illustrates a general tendency of users to doubt that their data will be protected:

I think there are loopholes and problems, and that data is not protected adequately.

A1; A5; A6; A7; A8; A9

Most (6/10, 60%) participants expressed resignations. Although they were concerned about their privacy, they also recognized that if they wish to participate in web-based activities or use certain apps, they must accept the terms of use and may lose control over their data. Many eventually decide to use an app, while maintaining some degree of privacy awareness:

Let me put it like this: I have a rather low data protection awareness. I ignore who can process my data.

A1; A2

I would describe my data protection awareness as not consistent enough to protect my data.

A3; A5

I take care of my data. Nevertheless, I think that I cannot really influence or intervene and determine who gets my data. As soon as I download and use an app, I have to agree to the terms of use.

A6; A7

However, some (2/10, 20%) participants expressed no concerns at all. A prominent issue is confidence in the manufacturer (in this case, Amazon).

The way I see it, as long as I feel sure, I will use the device; if trust is no longer there, then I think that I will no longer use the device.

A6

I have great confidence in Amazon that my data will be stored securely.

A10

In summary, our results show that most (8/10, 80%) participants have privacy concerns about using a smart speaker and are not sure what happens to their data. However, the participants did not fear monetary loss or reputational injury. We found that participants were willing to suppress their own data protection concerns to use the device and justified putting aside any lack of trust in the provider.

#### mHealth App

In the group using the mHealth app, participants’ views on privacy and data protection concerns were split—half (5/10, 50%) of the participants said that they were not worried or concerned about the privacy of their personal data:

They cannot do much with my data anyway because I am an ordinary person. [...] what I use or look at or, how should I say it, [...] that is what every human being does, to put it like that.

B1

No. I’ve got nothing to hide.

B5

Not at all, actually. [...] And I have nothing to hide. But I am not afraid that this will end up anywhere.

B9

In contrast, (4/10, 40%) of the participants were concerned about data protection and privacy:

Yes, I have become very concerned. [...] Because the different sites are obviously not safe and a lot of data is collected about you, you do not know anything about it. Of course, it is great to have computers here, if you get a lot of information, but there is also a certain potential to become dependent on them, and as I said, I see a problem with surveillance and abuse.

B6

The most frequently mentioned concerns include surveillance, abuse, and use of your data against you:

Partly yes, but I mean I know what I can write about on my smartphone and what I cannot say. [...] I think you have to have restrictions about that. [...] I am just careful what I [...] write. [...] What would be on a postcard, you would write like that, I would say.

B10

Overall, our results show that participants vary in terms of their data protection concerns and how they engaged with data privacy, specifically with regard to using a smart speaker or an mHealth app.

#### Smart Speaker Versus mHealth App

Our results indicate that attitudes vary depending on the device and how information is captured or entered. As expected, participants were most sensitive to personal data. Somewhat surprisingly, our results indicate that participants are more concerned about personal speech recorded by a smart speaker than about personal health data entered into an mHealth app.

### Data Misuse

To better understand the degree to which participants’ concerns about data misuse are justified, we inquired about the perceived ramifications of misuse of data collected via the smart speaker or eHealth app.

#### Smart Speaker

Most (9/10, 90%) participants testing smart speakers associate smart speaker data misuse with personalized advertisements. Some have linked it to profiling, monetary loss, and data loss:

I think that my data will be used for advertisement. I mean that they sell my data to agencies to show me ads at the right time.

A7; A2

They could take money from my bank account. Alternatively, they will spam me with e-mails or ads.

A4; A10

#### mHealth App

Of the 7 participants who reported believing that their data were sold to unknown third parties, 3 (43%) assumed that their data were stored on unknown servers by companies such as Google. A few (3/10, 30%) participants had never considered this issue:

They trade our data, the data is sold, and that is well known. And yes, it is used to send advertisements to advertising companies.

B8

The data is just sitting around in some archive. Or someone can buy it. No idea. I have not really thought about it.

B4

Some (2/10, 20%) participants raised concerns about potential negative implications for existing insurance policies or apps for new insurance policies because of chronic or serious illnesses:

Yes, I do not see any problems there now, because my health is good. I can well imagine that others might have a problem because they think if everyone knows that I have trouble taking out an insurance policy or get offered worse conditions, but theoretically this is already the case.

B3

No, not really. I no longer have a problem with any health insurance companies at my age. I am privately insured so of course nothing will change. And I do not need to take out large insurance policies anymore, but if I were younger, I would be much more aware of the fact that the data might be misused.

B6

These statements indicate a much deeper understanding of the potential implications of data abuse in the health care context, including direct monetary damage. However, the risks described did not result in grave concerns or technology rejection among the respondents.

### Termination of Use

No participant in either group stopped using the technology or intended to stop using the technology after the test because of data privacy concerns, and we observed no increase in data privacy concerns over time. Across the board, participants assumed that data were collected to enable personalized advertisements, which did not represent a salient enough risk to participants to motivate the termination of use.

#### Smart Speakers

Participants testing smart speakers reported several data privacy issues that would hypothetically motivate them to stop using it, including privacy violations, data leaks, and eavesdropping:

I would stop using it if I myself were affected, such that people could openly access my data.

P5; P7; P9

If I suddenly got advertising/promotional mails or phone calls, I would not use it anymore.

P1; P3; P4; P8

However, such hypothetical concerns and risks did not motivate participants to discontinue using technology. Rather, participants would have to experience an incident personally to trigger actual termination of use.

#### mHealth App

Participants testing the mHealth app reported that no data privacy issues would hypothetically motivate them to stop using the app:

No, not at all.

B3

Not significantly.

B9

Interestingly, none of the participants were seriously concerned about data privacy and the protection of their health data when using the mHealth app.

## Discussion

### Principal Findings

This study aimed to determine whether data privacy is perceived differently for different data-intensive technologies. By comparing 2 groups of mature adult users, we assess how the impact of privacy concerns, such as data protection concerns and risks, on technology use and discontinuation differs depending on the technology.

In both user groups, none of the participants stopped using the technology, despite privacy concerns. This is in line with the widely discussed privacy paradox [36], which states that individuals engage with technology even when they associate it with potential privacy loss issues. This phenomenon has been observed in social media, e-commerce, and mobile apps [45,65,66]. A likely driver of this paradoxical behavior is that the perceived immediate benefits of using a technology outweigh its potential, hypothetical, future risks [67]. Our results also indicate that this applies to health data privacy risks as well. In both user groups, participants expressed widespread resignation that choosing to use the technology comes at the cost of a greater risk of potential loss of data privacy.

Surprisingly, our results also show that people do not associate greater risk with personal health data collected by an mHealth app than with spoken words recorded by smart speakers. Because personal health data are highly sensitive and provide deep insights into individuals, one would expect users to be more concerned about protecting their privacy [68]. One possible explanation is that discrete data entered manually, consciously, and willingly, such as health status data in an mHealth app, are easier to control than impromptu utterances spoken all day long in a private setting. Regardless, our results call into question whether users of information technology, who require personal information distinguish between the data types collected by various devices and whether users fully understand the financial and personal ramifications of data abuse beyond personalized advertising.

In contrast with the mHealth app, which only passively receives data entries, a smart speaker may be perceived as invasively and nontransparently intruding into the private space. Indeed, several smart speak testers referred to the device’s constant *listening* for the activation keyword as *spying* in the pretest interviews. However, no participants used this term in the posttest interviews after the 6-month test phase. This may indicate that they perceived greater risk to their privacy in the preadoption than in the postadoption phases, as they are commonly referred to in technology adoption research [69,70].

Our results provide initial indications of a potential decline in the perceived fear of loss of private health data with increasing age. Several older participants stated that no one would be interested in their health data; therefore, privacy breaches posed no threat to them. Further research is needed to discern consistent patterns of age-related differences.

### Implications for Research

This exploratory research contributes to research on how data privacy concerns influence the intended and continuous use of data-intensive technologies, specifically mHealth apps and smart speakers. Investigations into the specific perceptions and resulting behaviors of mature adults in privacy research are scarce, and scholars still have only a rudimentary understanding of how older people’s engagement with technology is influenced by privacy-related issues and concerns. This is especially important, as mHealth apps and voice-activated assistants gain increasing importance in providing health care–related services to mature and older adults [71].

Our findings have 4 major implications for research. As our findings are based on an exploratory qualitative research method, their implications do not aim to be representative of all mature adults but provide a basis for further research to investigate quantitatively.

First, our findings indicated that mature adults’ self-reported privacy concerns did not directly influence their actual use behavior once they had adopted a data-intensive technology. This finding is consistent with the privacy paradox [36]. Participants frequently entered a state of resignation, acknowledging that choosing to use the technology requires them to accept the potential loss of data privacy.

Second, our results indicated that mature adults sometimes view their personal data indiscriminately. Even when participants were aware of the risks of data misuse, they articulated that the risks did not affect them personally or would not manifest themselves in their case. The risk that participants mentioned the most was receiving more personalized advertisements. In fact, participants valued protecting their general personal data more than protecting their personal health data, positing that they were not worthwhile targets of health data theft. This is concerning, especially considering recent health-related data hacks [72].

Third, our results pointed to age as a moderating factor in the perception of data privacy, risk assessment, and the subsequent application of privacy calculus. Several older participants articulated that their data privacy–related risks were low because of their age. Lee et al [73] showed that the tendency to discount future risks was prevalent among younger people. Our study indicated that this influence may be more substantial for older people who, according to our study, lack motivation to engage with how their personal data will be treated and are, therefore, more willing to disclose their personal data.

Finally, somewhat surprisingly, we found that data type did not significantly affect how participants perceive data privacy issues. We expected that participants would be more concerned about protecting the privacy of their personal health data than about protecting more general personal information. However, participants initially considered all personal data as equally worthy of protection, possibly insufficiently understanding the ramifications of data abuse and not reliably distinguishing between different types of data processed through different devices. Further research is needed on the role of data types in technology users’ privacy calculus, as current privacy models do not distinguish among different types of data or consider individuals’ perceptions of different types of data.

### Limitations and Further Research

This study is exploratory in nature; therefore, our results are not generalizable to all mature adults and all data-intensive technologies. Furthermore, all participants were recruited in the south of Germany and thus shared a certain cultural background. As with every qualitative study, this study is subject to potential bias from the research team and potentially influenced by social desirability bias among participants.

Our findings suggest several avenues for further research. Specifically, we call for further research on how the act of *resignation* manifests in users’ privacy calculus as an acceptable price to pay for using a certain technology. Further research is also needed to understand what drives people to value certain types of personal data more than others, which, in our case, is valuing general personal data used to personalize advertising more than personal health data, which can be misused with grave consequences. Finally, a cross-generational study is required to assess the influence of age on data privacy concerns and technology adoption.

### Conclusions

Research on how mature adults’ data privacy concerns influence their use of data-intensive technology is scarce, despite reports of data hacks and leaks and eavesdropping on prominent technologies and the sensitive nature of personal health data. The results of our exploratory research analyzing interviews with 20 mature adult users of data-intensive technologies reveals that although participants self-reported initial data privacy concerns, they did not value the risks high enough to discontinue using the data-intensive technologies in focus. Rather, they expressed widespread resignation that choosing to use the technology means accepting the risk of loss of data privacy. This fatalistic surrender, combined with evidence that participants valued their general personal data more than their personal health data, is a cause for concern about the security of personal data among technology users of this generation.

## Abbreviations

mHealth: mobile health
RQ: research question
